# Auscultation and Percussion

**Published:** 1854-11

**Authors:** 


### BIBLIOGRAPHICAL NOTICES.

*Auscultation and Percussion.* By JOSEPH SKODA. *Translated from the Fourth German Edition.* By W. O. MARKHAM, M. D., Assistant Physician to St. Mary's Hospital. Philadelphia, Lindsay & Blakiston, 1854.

*A Practical Treatise on the Diseases of the Lungs, Heart and Aorta, including the principles of Physical Diagnosis.* By WALTER HAYLE WALSHE, M. D., Prof. of Clinical Medicine in University College, London. *Second Edition.* London, Walton & Maberly, 1854.

*A Clinical Introduction to the practice of Auscultation and other modes of Physical Diagnosis in diseases of the Lungs and Heart.* By H. M. HUGHES, M.D. *Second American from the Second English Edition.* Philadelphia, Blanchard & Lea, 1854

[Continued from page 608.]

The *râles* are divided by Skoda into
Vesicular râles,
Consonating râles,
Dry crepitant râle with large bubbles,
Indeterminate râles,
Râles accompanied by amphoric resonance and metallic tinkling.

Skoda's *vesicular râle* is the "râle crepitant humide" of Laennec. It includes the crepitant and subcrepitant râle of the authors of the present day, and is produced by the presence of blood, mucous, serum, etc., in the smaller bronchial tubes or in the air-cells. When very fine, or crepitating, it was considered by Laennec to be characteristic of the first stage of pneumonia. That this is not the case, has already been shown by the observations of Cruvelhier and Chomel; Skoda adds his high authority to deny its pathognomonic character, and supposes it simply to denote the presence of fluid in the air-cells.

The *consonating râles* are produced in consolidated tissue, under the same conditions which give rise to bronchial respiration and thoracic voice. They are probably the "râles cavernuleux à timbre clair" of some of the French auscultators.

*The râles accompanied by amphoric echo and metallic tinkling* are met with in pneumothorax and in large cavities. They may sometimes, Skoda states, be observed in cases, in which no communication exists between the pneumothorax and the bronchial tubes, and may themselves excite distinct metallic tinkling.

Under the name of *indeterminate râles* all those rhonchi are described which offer no special indications respecting the condition of the lung-tissue. The same objection may, we think, be urged against the introduction of the term "indeterminate râle" as against that of "indeterminate respiration."

It will be seen from this division of the râles, that Skoda has retained a râle, which is for the most part excluded from the list of the auscultatory phenomena—Laennec's dry crepitating râle with large bubbles; whilst the cavernous râle or gurgling he entirely discards. This *dry crepitant* râle or "craquement," (not the crackling or "craquement" in the sense of Fournet) does not seem to have been distinguished since the time of Laennec; Skoda, although he does not attach much diagnostic value to it, yet testifies to its existence: he believes that it is caused by simple distension of the walls of the air cells during inspiration.

The *cavernous râle* or *gurgling* is, according to Skoda, simply a consonating râle, and as little pathognomonic of a cavity as cavernous respiration or pectoriloquy. Now, nowhere does Skoda's tendency to simplify show itself more strongly, and in no particular would his nomenclature, if introduced, prove of more service. Author after author has demonstrated, that the gurgling sound, unless it be accompanied with a metallic ringing, is not only prdouced in cavities, but frequently also in enlarged bronchi, and may even be heard over consolidated tissue. The accurate distinction between it and the mucous râle all admit to be, judging from the sound alone, impossible, yet the term "cavernous râle" or "gurgling" as pathognomonic of a cavity, is retained in almost every work on Auscultation. Laennec himself states that the only sign distinguishing the cavernous from the mucous râle is, that the bubbles of the former are louder and more abundant than those of the latter, and are heard in a circumscribed space. The number and the size of the bubbles surely depend only upon the force of the respiration, whilst no one can deny that all râles appear louder in consolidated tissue, whether we admit or not as the cause, Skoda's theory of consonance.

The *dry râles* of Laennec, Skoda does not describe as a variety of the rhonchi, but as sonorous, whistling, and hissing sounds. The "craquement" of Fournet, the "*crackling*" of the English writers, which is clinically connected with the softening of tubercle, Skoda does not separate from the ordinary moist râles, although he strangely admits it to be a dry sound. Its mechanism, as well as the distinction between its humid variety and the subcrepitant râle, is as yet far from being satisfactorily demonstrated. Thompson, in his recently published lectures on Pulmonary Consumption, lays great stress on its existence as an evidence of tubercle previous to the commencement of the stage of softening. Barth and Roget* describe it as a sound separate from the moist râles, but admit that it may easily be confounded with them. Walshe, in his new edition, retains the crackling as a distinct sound, but offers no explanation of its mechanism, nor does he, in our opinion, dwell sufficiently on the difficulty of a differential diagnosis between the moist crackling and the subcrepitant.

Fournet's "*bruits de froissement pulmonaire*," Skoda dismisses in the following summary manner:

> "Several varieties of murmurs are included under the denomination of froissement, all of which are supposed to have this character in common, that they give to the person observing them the impression that he can see the lung-tissue struggling with force and noise against some obstacle to its distension. I have never yet met with such a struggling sound, and shall abstain from offering any opinion upon it until its existence shall have been clearly demonstrated."

As the diagnosis of cardiac affections turns greatly upon a correct appreciation of the physiological actions of the heart, Skoda commences the chapter on the auscultatory phenomena of the *organs of circulation*, with a consideration of the impulse and of the sounds of the heart. The theory he adopts of the cause of the *impulse* is the one known as the recoil-theory of Gutbrod.

> "It is a well-known physical law, that when a fluid escapes from a vessel, the equality of pressure produced by the fluid on the walls of the vessels is lost, for there is no pressure at the opening whence the fluid escapes: but at that part of the vessel, which is opposite to the opening,

* Traité d'Auscultation, p. 173.

the pressure is still exerted. This pressure it is which set Segner's wheel in motion, and produces the recoil of fire-arms, &c. By contraction of the ventricles, the pressure which the blood exerts upon the walls of the heart, opposite to the opening whence the stream escapes, causes a movement of the heart in a direction contrary to that of the stream of blood; and by this movement the impulse of the heart against the walls the thorax is produced." (p. 183.)

As further causes of the impulse, Skoda mentions the lengthening of the aorta and pulmonary artery during the systole, also the change of form, and the rigidity the organ assumes, during this action of its fibres. The contraction of the papillary muscles he does not believe to take any part in its production; nor does he attribute much diagnostic value to its force. A healthy heart, when excited, will produce a strong impulse, whilst an hypertrophied heart may sometimes beat so calmly as to render the impulse almost imperceptible. The beat of a healthy heart is, however, never felt over more than one or at most two intercostal spaces.

The different theories of the causes of the *sounds* of the heart are next elaborately reviewed by Skoda. He has based his own theory of their production mainly on clinical and pathological observations. The *first* sound, he writes, is produced by the impulse of the blood against the mitral and tricuspid valves, and by their sudden tension during the systole, also

"by the impulse of the heart against the walls of the thorax. A blow struck with the finger, or with the apex of the heart firmly compressed against the inner surface of the thoracic walls, produces a clinking (Klirrend) sound, which differs in no particular from the ordinary first sound of the heart. If a part of the walls of the heart be separated from the thoracic walls during the ventricular diastole, but strike against them during the systole; or even if the heart beat against some other part of the thoracic walls during its systole than that beneath which it lay during the diastole, still in either case a clinking sound or one exactly resembling the ordinary first sound of the heart will be produced, for the heart's substance becomes firm during its systole."

Yet all these causes, he admits, are not sufficient to explain satisfactorily the cause of the impulse in every instance.

The explanation of the *second* sound Skoda regards to be yet more difficult than that of the first sound. It does not, he thinks, always originate in the ventricles, since it is sometimes heard more distinctly over the base than over the apex of the heart. It is most frequently the transmitted arterial sound; occasionally it may arise, from the impulse of the blood against the walls of the ventricle, during the recedence of the heart's point from the surface after the systole; an opinion to which Walshe strongly inclines. The second sound heard over the aorta and pulmonary artery is produced by the closing of the semilunar valves. To the reduplication of this sound Skoda assigns but little diagnostic importance, but he attaches much more value to the intensification of the second sound over the pulmonary artery. The sounds of this artery are best listened to, not at their seat of production, but to the left of the sternum between the second and third ribs. Normally the second sound heard here is more intense than the second sound at the apex of the heart, but it is of equal force with the one audible over the aorta. In affections of the mitral valve, and especially in its constriction, the pulmonary artery and veins become engorged, and the right ventricle has to drive the blood with greater force through the vessels. The pulmonary artery, thus distended, presses, during the diastole, the blood with greater force back on the valves of the heart, and an increased sound is the result. Thus an accentuated second pulmonary sound becomes, Skoda assumes, an important diagnostic sign of mitral disease, or else of hypertrophy of the right side of the heart. He even goes so far as to state, that a blowing sound at the mitral orifice, unless it coincide with a distinct second sound over the pulmonary artery, denotes simply roughening of the surface of the valve, but never insufficiency or constriction. That under these circumstances an increased distinctness of the sound frequently occurs, no one can question, but both Walshe and Stokes characterize this exclusive diagnostic sign proposed by Skoda, as "extremely hazardous."

The *endocardial murmurs* Skoda refers to the friction of the blood against the walls of the heart, which occurs either in organic change, or without any appreciable alteration of its structure. How this friction causes blowing sounds is unexplained; occasionally they may be produced by the rapid flow of a small stream of blood moving in an opposite direction.

"I would add, that murmurs may also be produced within the heart's cavities, by the rapid flow of a small stream of blood against blood that is quiescent, or moving less rapidly, or in an opposite direction to it. The fact of a murmur being created when a small stream of any fluid, as water, blood, etc., is forced rapidly against a fluid at rest, may be demonstrated by a direct experiment." (p. 244.)

The opinion that blowing sounds originate, in some diseases, from a watery state of the blood, Skoda considers hypothetical, nor does he admit Andral's statement that they may be produced by general plethora. The timbre of these endocardial sounds, to which Hope and Gendrin attributed such importance, he regards (and Walshe agrees with him in this) of but slight value for diagnostic purposes:

"It is vain to attempt to distinguish too particularly between the different kind of murmurs; indeed it matters little, as respects any conclusions which may be deduced from our observations, whether the murmurs are of a blowing, sawing, or rasping character." (p. 246.)

If these blowing sounds be heard synchronously with the systole over the mitral and tricuspid valves they denote insufficiency; if over the aorta and pulmonary artery, constriction of the orifice; whilst a blowing sound occupying the diastole, and most intense over the aorta, is produced by insufficiency of its valves. The murmurs arising in the course of other diseases and unconnected with organic affection of the valves, are not, we think, sufficiently fully considered by Skoda.

The *pericardial* sounds are sometimes the easiest, sometimes the most difficult, to distinguish of all the sounds connected with the circulatory apparatus. These pericardial friction sounds were not known to Laennec. Their value was first pointed out by Collins: they were subsequently more accurately described by Stokes in his valuable paper on pericarditis. Bouillaud (Maladies de Cœur) speaks confidently of their easy distinction from the endocardial sounds, and describes three distinct varieties—the rustling sound, "bruit de frôtement;" the new leather sound, "bruit de cuir neuf;" and the grating sound, "bruit de raclement." Graves, Stokes, and Walshe regard them as generally readily recognizable, but admit that cases may occur in which the distinction between a pericardial and endocardial sound becomes a matter of great difficulty. Skoda states that no pericardial sound can occur which may not resemble an endocardial murmur, and denies that they are possessed of a pathognomonic, superficial and extended character.

"According to my own experience, indeed, there is no kind of endocardial murmur, with the exception of the whistling, which may not be imitated by a friction sound of the pericardium, and no pericardial murmur which may not resemble an endocardial murmur.

"In my opinion friction-sounds of the pericardium cannot be distinguished from internal murmurs of the heart, by being more superficial, more extended and more diguised than these. A sound proceeding from a solid body, when strong and clear, appears to arise at its surface; but when of an opposite character, to come from some distant part of it. Thus, murmurs arising within the heart may appear quite superficial in consequence of their being inordinarily loud, clear, high and hissing, and may be audible over the whole region of the heart and even beyond it; on the other hand the friction sound of the pericardium may be very weak and muffled, and thus appear to arise remotely." (p. 253.)

The only sign, according to Skoda, by which a pericardial friction sound may be distinguished from an endocardial blowing sound is, that the latter corresponds pretty exactly to the rhythm and sounds of the heart, while the former, on the contrary, follows upon the movements of the heart. Stokes mentions, as reliable signs of distinction, the limitation of the peri cardial sounds to the region of the heart, their increase by pressure, and their changeableness in seat and intensity. Walshe lays great stress on the last-named sign, as well as on the want of synchronism with the heart's action, and on the superficial character of the sound; on the value of pressure (first noticed by Sibson as a diagnostic mark) he has of late somewhat modified his opinion :

"I leave this clause as it stood in the first edition, although I confess I daily lose faith in the efficacy of pressure as a positive means of distinguishing intra-cardiac and pericardial murmurs. There are several reasons, it appears to me, why implicit trust cannot be placed in the effects of pressure. First, an indubitable friction-sound may be weakened by pressure, if carried beyond a certain amount; secondly, a pericardial rubbing-sound may be sometimes induced by pressure, where the pericardium is free both from exudation matter and vascularity; thirdly, a true endocardial murmur may be increased in thin persons with flexible chests by regulated pressure—possibly not endocardial murmurs of all sites and all rhythms, but certainly systolic basic murmurs. The increase in intensity seems to be sometimes merely the effect of improved conduction." (Walshe, p. 262.)

The blowing sounds heard in the *arteries* may have their origin in a disease of the heart, or in a roughening of the internal membrane of the artery, but may also arise when the coats of the latter are perfectly normal. A blowing systolic murmur, Skoda has often heard in healthy individuals, "when their heart's action was increased, and particularly if their cervical muscles were at the same time put upon the stretch." A blowing sound, he states, is further produced when small arteries are widened, a circumstance which, according to Kiwisch, would account for the placental bruit, which this author refers to the epigastric artery. Whether these arterial murmurs be caused by friction of the blood against the walls of the arteries, or simply by the vibration of their coats, Skoda does not decide.

The *continuous murmurs* heard in the *veins*, the "bruit de diable" of Bouillaud, were at first supposed to be of arterial origin. Skoda inclined formerly to this opinion; he now admits their venous seat, although he considers the cause of their production as unsettled. They may, he states, be observed in the young and healthy; and are, therefore, not the result of spanæmia. Dr. Hughes considers them connected with pressure, either directly or indirectly applied; and dependent upon partial obstruction to the quickened flow of the blood through the jugular veins. They are always, he thinks, connected with a watery state of the blood. Walshe adduces several explanations of this phenomenon, yet admits that its real mechanism is obscure. Clinically, however, he regards the venous murmur as indicative of hydræmia; a view borne out by the researches of Andral.

"I would sum up on this matter, clinically, by saying that a state of the blood in which the venous blood becomes slightly soniferous, is not incompatible with apparent health; but that intense, readily audible and diffused venous murmur, is characteristic of those morbid conditions of the fluid called hydræmia, anæmia and spanæmia." Walshe, p. 283.

With this extract we must conclude our notice of the works with the names of which our review was headed. The succeeding chapters, forming the second portion of Prof. Skoda's work, treat of the normal and abnormal phenomena of the thoracic and abdominal organs, discoverable by the aid of percussion and auscultation. Here we find the diagnostic applications of the general principles, discussed in the first part of the work, with an enumeration of many valuable facts, furnished by the extensive clinical experience of the distinguished author. We will not attempt to consider this portion of the work critically, but recommend every line of it most strongly to our readers as worthy of the most careful perusal. It has, indeed, in this review, been mainly our object to discuss those views on the general principles of physical diagnosis, and on the production of sounds with which Skoda's name is everywhere associated, and to point out the differences between him and other distinguished observers. In taking leave of our author, we may be permitted to express it as our honest belief, that as a work replete with original thought and close observation, Skoda's treatise stands only next in importance to the great work of Laennec, and Dr. Markham has placed the profession under obligations, by his excellent translation of so valuable a contribution to medical literature.

An analysis of the second edition of Prof. Walshe's treatise we have attempted, merely as far as it concerned our object in view. A more minute examination of this valuable work, which, as a treatise on thoracic affections, may safely be pronounced unrivalled, we reserve for a future occasion. In this edition, new diseases, previously omitted, have been introduced; the anatomical characters of each morbid state added; and the sections on diagnosis and treatment materially extended; so that instead of a new edition, Prof. Walshe has, in reality, presented us with a new and most complete treatise on the diseases of the chest.

The second edition of Dr. Hughes little work, does not differ materially from the first. Amongst the new matter introduced, we observe a note on the cause of emphysema, and an extension of the chapter on Anæmic Murmurs. The work is written in a plain, easy style, and will, we doubt not, be a popular manual for those wishing to acquire a knowledge of the elementary principles of Percussion and Auscultation.

